# Accelerating access to quality TB care for pediatric TB cases through better diagnostic strategy in four major cities of India

**DOI:** 10.1371/journal.pone.0193194

**Published:** 2018-02-28

**Authors:** Neeraj Raizada, Sunil D. Khaparde, Virender Singh Salhotra, Raghuram Rao, Aakshi Kalra, Soumya Swaminathan, Ashwani Khanna, Kamal Kishore Chopra, M. Hanif, Varinder Singh, K. R. Umadevi, Sreenivas Achuthan Nair, Sophie Huddart, C. H. Surya Prakash, Shalini Mall, Pooja Singh, B. K. Saha, Claudia M. Denkinger, Catharina Boehme, Sanjay Sarin

**Affiliations:** 1 Foundation for Innovative New Diagnostics, New Delhi, India; 2 Central TB Division, Government of India, New Delhi, India; 3 Indian Council of Medical Research, New Delhi, India; 4 State TB office, Govt of NCT, Delhi, India; 5 New Delhi TB Centre, New Delhi, India; 6 Lady Hardinge Medical College and assoc Kalawati Saran Children’s Hospital, New Delhi, India; 7 National Institute of research in Tuberculosis, Chennai, India; 8 World Health Organization, Country Office for India, New Delhi, India; 9 McGill University, Montreal, Canada; 10 Intermediate Reference Laboratory, Hyderabad, India; 11 Intermediate Reference Laboratory, Kolkata, India; 12 Foundation for Innovative New Diagnostics, Geneva, Switzerland; Indian Institute of Technology Delhi, INDIA

## Abstract

**Background:**

Diagnosis of TB in children is challenging, and is largely based on positive history of contact with a TB case, clinical and radiological findings, often without microbiological confirmation. Diagnostic efforts are also undermined by challenges in specimen collection and the limited availability of high sensitivity, rapid diagnostic tests that can be applied with a quick turnaround time. The current project was undertaken in four major cities of India to address TB diagnostic challenges in pediatric population, by offering free of cost Xpert testing to pediatric presumptive TB cases, thereby paving the way for better TB care.

**Methods:**

A high throughput lab was established in each of the four project cities, and linked to various health care providers across the city through rapid specimen transportation and electronic reporting linkages. Free Xpert testing was offered to all pediatric (0–14 years) presumptive TB cases (both pulmonary and extra-pulmonary) seeking care at public and private health facilities.

**Results:**

The current project enrolled 42,238 pediatric presumptive TB cases from April, 2014 to June, 2016. A total of 3,340 (7.91%, CI 7.65–8.17) bacteriologically confirmed TB cases were detected, of which 295 (8.83%, CI 7.9–9.86) were rifampicin-resistant. The level of rifampicin resistance in the project cohort was high. Overall Xpert yielded a high proportion of valid results and TB detection rates were more than three-fold higher than smear microscopy. The project provided same-day testing and early availability of results led to rapid treatment initiation and success rates and very low rates of treatment failure and loss to follow-up.

**Conclusion:**

The current project demonstrated the feasibility of rolling out rapid and upfront Xpert testing for pediatric presumptive TB cases through a single Xpert lab per city in an efficient manner. Rapid turnaround testing time facilitated prompt and appropriate treatment initiation. These results suggest that the upfront Xpert assay is a promising solution to address TB diagnosis in children. The high levels of rifampicin resistance detected in presumptive pediatric TB patients tested under the project are a major cause of concern from a public health perspective which underscores the need to further prioritize upfront Xpert access to this vulnerable population.

## Background

Tuberculosis is an important cause of childhood morbidity and mortality. It is estimated that around 10% of the 10.4 million global incident TB cases and 250,000 of the 1.7 million TB deaths in 2016 were amongst children (<15 years). Globally, children accounted for only 6.9% of the new cases that were notified in 2016 [1]. Under and delayed diagnoses of TB in children remains an obstacle to effective management of childhood TB due to which cases often remain underreported [2–3]. In high TB burden settings, it is estimated that childhood TB contributes to 15–20% of all TB cases and is one of the leading causes of childhood mortality [4]. India, which is among the highest TB and DR-TB burden countries globally, notified 76,745 childhood TB cases in 2016, accounting for only 5% of the total notified TB cases in the country [5].

Diagnosis of TB in children is challenging, is largely based on triad of, a) positive history of contact with a TB case, b) clinical and radiological findings and c) tuberculin skin test [5–9]. This is often without microbiological confirmation due to practical concerns in obtaining a sputum specimen, necessitating collection of alternate types of specimen in children [10–11]. Additionally, clinical diagnosis of TB is challenging in children, as signs and symptoms of TB in children can be very non-specific and similar to other common childhood chest infections [2, 10]. Limited availability of high sensitivity rapid diagnostic tests that can be applied with a quick turnaround time often leads to microbiological confirmation not being attempted [12, 13]. These diagnostic challenges and over reliance on clinical diagnosis limit the possibility of diagnosis of rifampicin-resistant TB (RR-TB) [14,15].

The WHO has recommended upfront Xpert MTB/RIF (Xpert) testing for the diagnosis of TB in pediatric presumptive pulmonary and extra-pulmonary (EPTB) cases [16–17]. Upfront rapid testing with Xpert assay, offers a promising solution to achieve the global objective of early and accurate detection of TB and rifampicin-resistant TB which is crucial for the timely initiation of accurate treatment in this vulnerable population [12,18]. Against this backdrop, the current project was undertaken in four major cities of India, namely Delhi, Chennai, Kolkata and Hyderabad, to address TB diagnostic challenges in the pediatric population, by offering free of cost upfront Xpert testing to pediatric presumptive TB cases. This project was initially undertaken in a pilot mode, from April- November, 2014, provided promising results [19]. However, this initial pilot did not address several aspects which were crucial to scalability and replicability of the intervention, including sustainability of the rapid turnaround time demonstrated in the initial phase over an extended period of time, impact of early diagnosis on TB care and mortality, etc. Further, some of the key findings observed in the initial pilot needed to be further validated on a larger cohort. We report here the larger project data from April, 2014 to June, 2016 and our experiences in rolling out the WHO recommendations in programmatic settings for pediatric presumptive TB cases in four cities of India and building the capacity of the health system to routinely offer upfront Xpert testing for all types of pediatric specimens including non-sputum specimens.

## Material and methods

The project was implemented in 4 major cities of India, namely Chennai, Delhi, Hyderabad and Kolkata, covering a population over 30 million, with the objective of providing all pediatric presumptive TB cases in these cities with upfront access to free of cost Xpert testing. High throughput Xpert laboratories were established under the project and were dedicated to the pediatric population. Using a hub and spoke model, one laboratory was established in each city which linked to various public and private sector health care providers in the city. While the exact size of pediatric population, including the burden of TB in children, in these large cities is unknown, geographic coverage of the project was ensured by means of rapid specimen transport linkages between the Xpert lab and linked public and private institutions/providers in these cities. All the presumptive pediatric TB and DR-TB cases referred from the collaborating clinics and hospitals (both public/private) were offered free of cost Xpert testing. Xpert test was performed on various types of specimens such as gastric aspirate/lavage (GA/GL), bronco-alveolar lavage (BAL), cerebrospinal fluid (CSF), sputum, lymph node aspirates, etc.

Children (age 0–14 yrs) presenting with signs and symptoms suggestive of TB to any of the public or private health facilities in the project areas between April 2014 to June 2016 were prospectively enrolled in the project. The different providers (both public and private) in the project cities were given an option of prescribing free of cost Xpert testing to the pediatric presumptive TB case identified by them in their health facilities. The health care providers were requested to refer the different types of patient specimens (sputum and non-sputum specimen) by leveraging rapid specimen transportation linkages for Xpert testing established as part of the project. Specimens were collected at the respective health facilities and transported to a centralized lab in each city for microscopy and Xpert testing. A number of sensitization workshops were organized with various mapped healthcare providers in the project cities to increase the project uptake. The specimen transportation linkages were planned across all four cities, taking into account feasible local transportation mechanisms acceptable to respective providers. The mechanisms deployed for rapid specimen transportation included use of commercial courier services and local volunteers whose incidental costs were reimbursed at a standard rate. The average cost of transportation covered under the project was one USD per specimen shipment received at the lab. The referring providers shared their contact details in the test request form. Specimens were subjected to smear microscopy using Ziehl-Neelsen (ZN) staining for comparison, with the first available specimen being tested on Xpert. A rapid reporting mechanism was established to ensure that all test results were promptly communicated back to providers utilizing e-mail and short messaging service (SMS).

Presumptive pediatric TB cases were defined as per the India’s Revised National TB Control Programme (RNTCP) guidelines [20,21]. This includes children presenting with fever and/or cough for ≥2 weeks, with or without weight loss or no weight gain, or showing symptoms suggestive of pulmonary and/or extra-pulmonary TB [18].

Bacteriologically confirmed TB (TB cases) were defined as having a pulmonary and/or extra pulmonary specimen positive for TB by smear microscopy, culture and/or Xpert MTB/RIF, or other WHO-approved rapid diagnostic test [18].

Rifampicin-resistant TB cases were defined as bacteriologically confirmed TB cases with indication of rifampicin resistance on one or more of the following assays: Xpert MTB/RIF, line probe assay (LPA) or phenotypic drug susceptibility testing (DST) [18].

For the purpose of analysis, presumptive TB cases with only pulmonary specimens positive for TB were classified as pulmonary TB cases and cases with only extra-pulmonary specimens positive for TB were classified as extra-pulmonary TB cases. Presumptive TB cases with both pulmonary and extra-pulmonary specimens positive for TB were classified as mixed TB cases.

**Fig 1:**
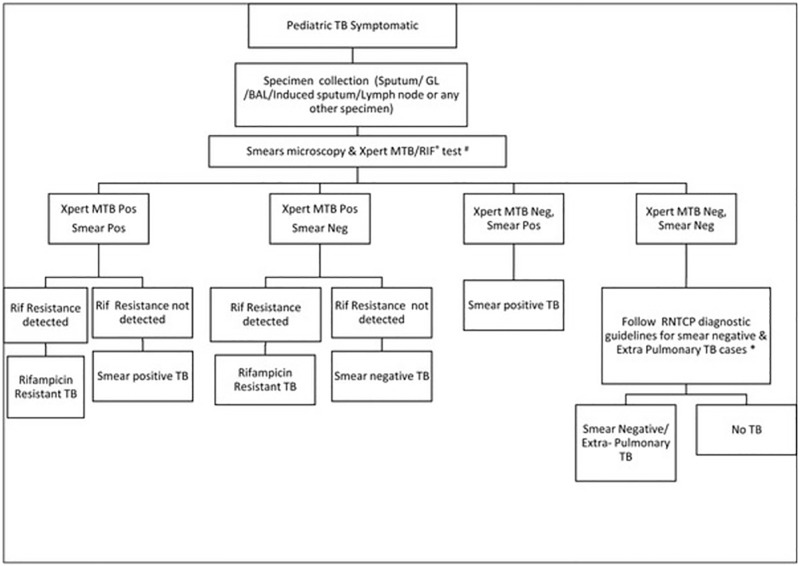
Project diagnostic algorithm.

Specimens were collected at the referral facilities which were linked with the project Xpert lab in the city. Sputum smear microscopy was conducted using RNTCP smear microscopy guidelines with all functional components of quality assurance (QA) in place [22]. Xpert testing was performed as per the project diagnostic algorithm (Fig 1). In cases where specimens were less than 1ml in volume, preference was given to Xpert testing ahead of smear microscopy in line with WHO recommendations [15–16]. For a given patient, whenever multiple types of specimens were available, all types of available specimen were tested. In case of ‘error’ and ‘no result’ test result on Xpert, a repeat test was performed on the remaining sample–buffer mix. In case of ‘invalid’ and ‘rifampicin resistance indeterminate’ test result, repeat testing was performed on a second specimen as per the WHO recommendation [16]. Sputum specimens were tested by adding buffer in 1:2 proportions as recommended by the manufacturer (Cepheid manufacturer instructions). For non-sputum specimens, standard operating procedures (SOPs) developed by RNTCP and WHO were adopted [23]. Confirmatory DST for diagnosed rifampicin resistant cases was performed on LPA and/or Xpert and/or culture DST. Confirmatory DST was performed either on the remnant specimen, or additional specimen, if available.

Treatment of all the diagnosed TB and rifampicin-resistant TB cases was initiated based on Xpert results in line with the project diagnostic algorithm as approved by RNTCP [24]. Any presumptive TB case receiving a negative result on Xpert and a positive result on smear microscopy were managed based on results of smear microscopy. Treatment of rifampicin resistant TB cases was based on initial Xpert rifampicin resistance results. Any case with rifampicin susceptible results on confirmatory DST was subsequently switched to appropriate regimen based on the decision of treating physicians.

Feasibility of Xpert implementation was assessed in terms of the ability of the assay to produce a valid result. The absence of a valid test result for any given assay performed was defined as a ‘test failure’ regardless of the underlying reason. The operational feasibility of offering Xpert testing to pediatric presumptive TB cases through a single lab in each of the 4 cities was assessed by analyzing the turnaround time (TAT) for specimen transportation, diagnosis and reporting of results to the providers.

### Data management

Data for all presumptive TB and DR-TB cases were collected from the RNTCP lab request form (Annexure I). The project was carried out under uncontrolled programmatic field conditions covering health facilities in the selected geographic areas. The data were fully anonymized and could not be traced back to specific individuals, or limited group of patients. Microsoft Excel 2013 was used for cleaning the data and data were analyzed using “R” software. All confidence intervals were calculated based on the binomial distribution with 95% probability interval (S1 Data). For analytical purposes, pediatric patients were categorized into 3 groups: 0–4 years, 5–9 years, and 10–14 years of age. Odds ratios were calculated to determine the statistical significance and relation between two variables.

### Ethical issues

Xpert testing for pediatric presumptive TB cases is an approved intervention under RNTCP. The current project was undertaken by FIND, after approval from and in collaboration with RNTCP. As such, the results presented here are our experience-sharing of implementing approved interventions in a programmatic setting within the existing accredited RNTCP TB diagnostic lab network. Since the observations described here are a part of implementation of approved interventions under RNTCP and a part of Standard of TB care in India, separate ethical clearance was not required.

## Results

Overall 42,238 pediatric presumptive TB cases and DR-TB cases were provided access to project interventions, across the four cities from April 2014 to June 2016. Of the 42,238 presumptive TB cases tested through the project, significantly higher proportion of cases came from public sector facilities (38047, 90.1%; CI 89.8–90.4) than private sector (4191, 9.9%; CI 9.6–10.2). Amongst them, the proportion of males (23,018, 54.5%) was higher than females (19,218, 45.5%). Similar proportions of presumptive TB cases from the three pediatric sub-age-groups were enrolled (Table 1). Overall, the median age of enrolled children was 7 years (IQR 3–11 years). Males were marginally, but not significantly younger (median 7, IQR 3–10) than females (median 8, IQR 4–11).

**Table 1:** Presumptive pediatric TB cases enrolled under the project and TB cases diagnosed on Xpert, stratified by age, gender, referring sector and prior history of TB treatment.

Variables		Number of patients tested	Number of diagnosed TB cases	Positivity rate, % (95% CI)	Proportion of total cases, % (95% CI)	Number of DR cases	Positivity of all TB cases, % (95% CI)	Proportion of total cases, % (95% CI)
	**Total**	42238	3340	7.9 (7.7–8.2)		295	8.8 (7.9–9.9)	
**Sub-Age group**	**0–4**	14082	681	4.8 (4.5–5.2)	20.4 (19.0–21.8)	42	6.2 (4.5–8.3)	14.2 (10.6–18.9)
	**5–9**	14045	751	5.4 (5.0–5.7)	22.5 (21.1–24.0)	66	8.8 (6.9–11.1)	22.4 (17.8–27.6)
	**10–14**	14111	1908	13.5 (13.0–14.1)	57.1 (55.4–58.8)	187	9.8 (8.5–11.2)	63.4 (57.6–68.8)
**Gender**	**Male**	23018	1293	5.6 (5.3–5.9)	38.7 (37.1–40.4)	119	9.2 (7.7–10.9)	40.3 (34.7–46.2)
	**Female**	19218	2047	10.7 (10.2–11.1)	61.3 (59.6–62.9)	176	8.6 (7.4–9.9)	59.7 (53.8–65.3)
	**Transgender**	2	0	0		0		
**Sector**	**Public**	38047	3034	8.0 (7.7–8.3)	90.8 (89.8–91.8)	270	8.9 (7.9–10.0)	91.5 (87.6–94.3)
	**Private**	4191	306	7.3 (6.5–8.1)	9.2 (8.2–10.2)	25	8.2 (5.5–12.0)	8.5 (5.7–12.4)
**Smear Status**	**Positive**	964	964	1 (0.1–1)	28.9 (27.3–30.4)	120	12.4 (10.5–14.7)	40.7 (35.1–46.5)
	**Negative**	38999	2219	5.7 (5.5–5.9)	66.4 (64.8–68.0)	160	7.2 (6.2–8.4)	54.2 (48.4–60)
	**NA**	2275	157	6.9 (5.9–8.0)	4.7(4.0–5.5)	15	9.6 (5.6–15.5)	5.1 (3.0–8.4)
**Prior H/O TB Rx**	**Yes**	1295	530	40.9 (38.2–43.7)	15.9 (14.7–17.2)	95	17.9 (14.8–21.5)	32.2 (27.0–37.9)
	**No**	40838	2808	6.9 (6.6–7.1)	84.1 (82.8–85.3)	200	7.1 (6.2–8.2)	67.8 (62.1–73.0)
	**NA**	105	2	1.9 (0.3–7.4)	0.1 (0.0–0.02)			

NA- not available

Of the 42,238 presumptive TB cases enrolled, 3,340 (7.9%, CI 7.7–8.2) pediatric TB cases were diagnosed. Among 3,340 pediatric TB cases, TB positivity in females (10.7%; CI 10.2–11.1) was observed to be almost two-fold higher than males (5.6%; CI 5.3–5.9); TB detection rates were similar in patients catered to by public and private sectors. Significantly higher TB positivity was observed in children in the 10–14 years age group (13.5%; CI 13.0–14.1) as compared to children in the 5–9 years age group (5.4%; CI 5.0–5.7) and 0–4 years age group (4.8%; CI 4.5–5.2). (Table 1)

Amongst the 3,340 pediatric TB cases diagnosed under the project, 295 (8.8%; CI 7.9–9.9) were found to be DR-TB cases. Higher levels of rifampicin resistance were observed in children in the 10–14 years age group (9.8%; CI 8.5–11.2) and 5–9 years age group (8.8%; CI 6.9–11.1) as compared to children in the 0–4 years age group (6.2%; CI 4.5–8.3). Similar levels of rifampicin resistance were observed in males (9.2%; CI 7.7–10.9) and females (8.6%; CI 7.4–9.9) and in patients from both public and private sectors facilities. (Table 1). Of the 295 cases, 95 (32.2%) had past history of TB treatment.

Of the 3340 TB cases detected under the project, 2534 (75.9% CI: 74.4–77.3) were pulmonary TB cases of which 210 (8.3% CI 7.3–9.4) were rifampicin resistant; 734 (22.0% CI 20.6–23.4) TB cases were extra-pulmonary TB cases of which 71 (9.7% CI 7.7–12.0) were rifampicin resistant. A total of 72 (2.2% CI: 1.7–2.7) TB cases had both pulmonary and extra pulmonary specimens positive for TB, of which 14 (19.4% CI: 12.0–30.0) were rifampicin-resistant. Of the 3340 TB cases, 185 (5.5%) were cases of TB meningitis of which 14 (7.6%) were found to be rifampicin-resistant. Confirmatory DST was performed on specimens showing rifampicin resistance. Overall findings indicate that majority of the valid TB-positive results on LPA were found to have concordant rifampicin results with Xpert. The detailed sub-analysis is on-going.

### Project turnaround time

Same-day turnaround for Xpert testing including specimen collection, transportation, testing and reporting was the norm (0 days (IQR 0–0 days)). Overall, the median days between reporting of results and treatment initiation was 3 days (IQR 1–6 days) and 8 days (IQR 4–16 days) for TB cases and DR-TB cases, respectively (Table 2).

**Table 2:** Project turnaround time: Median time between events in diagnostic cascade, median (IQR).

Variables	Days between collection and receipt	Days between receipt and testing	Days between testing and reporting	Days between reporting and treatment initiation (for those who initiated treatment after reporting)	Days between reporting and treatment initiation for DR TB (for those who initiated treatment after reporting)
**Total**	0 (0,0)	0 (0,0)	0 (0,0)	3 (1,6)	8 (4,16)
**Age**					
**0–4**	0 (0,0)	0 (0,0)	0 (0,0)	2 (1,4)	8 (2,115)
**5–9**	0 (0,0)	0 (0,0)	0 (0,0)	3 (1,6)	9 (4.5,16)
**10–14**	0 (0,0)	0 (0,0)	0 (0,0)	3 (1,6)	8 (4.3,15)
**Gender**					
**Male**	0 (0,0)	0 (0,0)	0 (0,0)	3 (1,6)	7 (4,12)
**Female**	0 (0,0)	0 (0,0)	0 (0,0)	3 (1,6)	9 (4,16)
**Transgender**	N/A	N/A	N/A	N/A	N/A
**Sector**					
**Public**	0 (0,0)	0 (0,0)	0 (0,0)	3 (1,6)	8 (4,15.5)
**Private**	0 (0,0)	0 (0,0)	0 (0,0)	1 (1,3)	9 (4.5,15.5)

N/A- not applicable

### Specimen wise analysis

For the 42,238 pediatric presumptive TB cases tested under the project, a total of 46,879 specimens were tested on Xpert and 41,918 specimens were tested using smear microscopy. The overall specimen-wise TB positivity rate on Xpert was 3,653/46,879 (7.8%; CI 7.6–8.0) and TB positivity rate on smear microscopy was 1,062/41,918 (2.5%; CI 2.4–2.7) (Table 3). TB case detection was more than threefold higher on Xpert as compared to smear microscopy independent of specimen types.

**Table 3:** Comparison of Xpert and smear positivity in different types of specimen.

Type of specimen	Total Specimens on Xpert	Total Positive	% (95% CI)	Rif cases	% (95% CI)	Total Tests on Smear	Smear Pos	% (95% CI)
**Total**	46879	3653	7.8% (7.6–8)	431	11.8% (10.8–12.9)	41918	1062	2.5% (2.4–2.7)
**Gastric Aspirate/ Lavage**	19703	1131	5.7% (5.4–6.1)	99	8.8% (7.2–10.6)	17462	243	1.4% (1.2–1.6)
**Induced Sputum/Sputum**	19178	1499	7.8% (7.5–8.2)	206	13.7% (12.1–15.6)	18658	685	3.7% (3.4–4)
**CSF**	3171	221	7.0% (6.1–7.9)	22	10.0% (6.5–14.9)	2282	0	
**Pus/FNAC/Lymph Node/Cervical Asp**	1332	519	39.0% (36.3–41.6)	72	13.9% (11.1–17.2)	998	87	8.7% (7.1–10.7)
**Pericardial Fluid**	74	8	10.8% (5.1–20.7)	0		52	1	1.9% (0–11.6)
**BAL**	1193	158	13.2% (11.4–15.3)	10	6.3% (3.3–11.7)	869	23	2.6% (1.7–4.0)
**Pleural Fluid**	1184	56	4.7% (3.6–6.1)	10	17.9% (9.3–30.9)	1109	8	0.7% (0.3–1.5)
**Ascitic Fluid**	297	9	3.0% (1.5–5.9)	1	11.1% (0.6–49.3)	214	1	0.5% (0–3)
**Endo-tracheal Secretion/ Tracheal Aspirate**	141	11	7.8% (4.2–13.9)	4	36.4% (12.4–68.4)	80	4	5.0% (1.6–13)
Others*	606	41	6.8% (5–9.2)	7	17.1% (7.7–32.6)	194	10	5.2% (2.6–9.5)

*Abscess, bone, Bone Marrow, Chyle Fluid, Cystic Fluid, Knee Aspirate, Liver Biopsy, Peritoneal Fluid, Pleural Biopsy, Right colonic ulceration, Serum, Skin Biopsy, Synovial Fluid, Thoracic swab, Tissue

First and second specimens were positive for 259 presumptive TB patients and 3 tests were positive for 28 children

A total of 46,879 different specimens were Xpert tested, of which 19,178 (40.9%) were sputum and 27,701 (59.1%) were various kinds of non-sputum specimens. Among the sputum specimens tested with Xpert, 1,499 (7.8%; CI 7.5–8.2) were found to be positive for TB and of the non-sputum specimens tested under the project the overall TB positivity on Xpert was 2,154 (7.8%; CI 7.5–8.1). Smear microscopy yielded fewer positive diagnoses compared to Xpert testing. Of the 41,918 different specimens subjected to smear microscopy, 18,658 (44.5%) were sputum specimens and 23,260 (55.5%) were non-sputum specimens. Among 18,658 sputum specimens, 685 (3.7%; CI 3.4–4.0) were smear positive, while among 23,260 non-sputum specimens tested on smear microscopy, 377 (1.6%; CI 1.5–1.8) were TB positive. (Table 3)

Higher TB positivity on Xpert were observed on Pus/FNAC/lymph nodes (39.0%; CI 36.3–41.6), followed by BAL (13.2%; CI 11.4–15.3) specimens. Further, as compared to smear microscopy, significantly higher TB positivity on Xpert testing was observed on pericardial fluid, tissue, tracheal aspirate and CSF. However, low TB positivity was observed on pleural and ascitic fluid specimens. (Table 3)

Specimen-wise analysis showed that of 3,653 specimens found positive for TB on Xpert, 431 (11.8%; CI 10.8%-12.9%) specimens were rifampicin-resistant. Of these, 225 (52.2% CI 47.4–57.0) rifampicin-resistant specimens were non-sputum specimens (Table 3). Of a total of 46,879 different specimens tested on Xpert, 40,215 (85.8%; CI 85.5–86.1) were pulmonary (sputum, induced sputum, BAL, tracheal aspirate & gastric aspirate) and 6,664 (14.2%; CI 13.9–14.5) were non-pulmonary specimens. Higher Xpert TB positivity was observed amongst non-pulmonary specimens as compared to pulmonary specimens (854, 12.8%; CI 12.0–13.7 vs. 2,799, 7.0%; CI 6.7–7.2).

### Yield of valid results on Xpert

**Fig 2:**
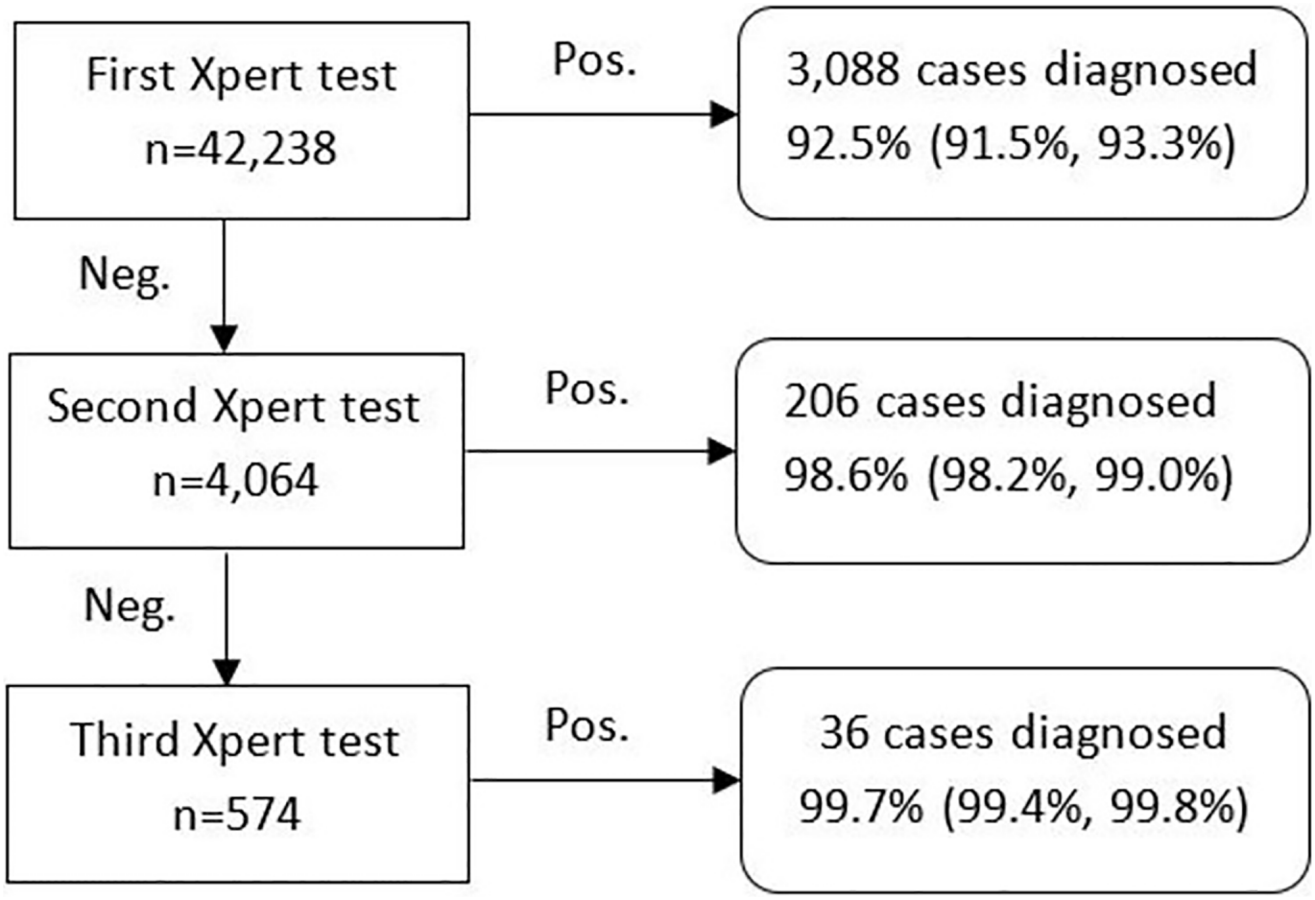
Incremental yields among first, second & third round Xpert testing.

Overall out of the 42,238 pediatric presumptive TB cases tested in the project, valid test results were provided to 99.7% of the cases by ensuring retesting of initial test failures. Of these, a single specimen was tested for 38,173 patients, 3489 patients had two specimens tested and 576 patients had three specimens tested. Overall, of the 3340 paediatric TB cases detected under the project 92.5%; (3,088, CI 91.5%-93.3%) were detected on the first Xpert test, and an additional 206 cases after the second Xpert test, with a cumulative positivity rate 98.6% (CI 98.2%-99.0%) and an incremental yield of 6.1% on a second Xpert test. Additional 36 cases were diagnosed after the third test, i.e. incremental yield of 1.1% (Fig 2).

### Treatment details on pediatric TB cases

Among 3,045 rifampicin-sensitive TB cases, 2,738 (89.9%; CI 88.8–90.9) patients were initiated on treatment, 60 (2.0%, CI 1.5–2.5) died, 118 (3.9%; CI 3.2–4.6) were lost to follow-up prior to treatment initiation and 6 (0.2%, CI 0.1–0.5) were ‘transferred out’ for treatment initiation to their respective TB project management units under RNTCP. Treatment initiation for a total of 123 pediatric TB cases (4.0%) could not be confirmed as these cases could not be traced. The treatment initiation rates were similar for male and females; however, significantly lower treatment initiation rates were observed in the 0–4 years age group as compared to the other pediatric sub-age-groups. Lower treatment initiation rates in this age-group in the project cohort was likely due to higher pre-treatment mortality and higher initial loss to follow up rate (Table 4). Similarly, among 295 rifampicin-resistant TB cases, 257 (87.1%; CI 82.6–90.6) cases were confirmed to be initiated on treatment. Of the remaining cases, 18 (6.1%; CI 3.8–9.6) died before treatment initiation and 14 (4.8%; CI 2.7–8.0) were lost to follow up prior to treatment initiation. The remaining 6 (2.0%; CI 0.8–4.6) could not be traced for treatment initiation verification and follow-up. The proportion of DR-TB cases initiated on treatment was significantly lower in 0–4 years age group as compared to other pediatric sub-age groups again due to higher rates of pre-treatment mortality and initial loss to follow-up. (Table 5)

**Table 4:** Treatment initiation status of bacteriologically confirmed rifampicin sensitive TB cases.

Variables	Total	Initiated treatment	% (95% CI)	Died	% (95% CI)	Lost to follow-up	% (95% CI)	Transferred Out	% (95% CI)	NA	% (95% CI)
**Overall**	3045	2738	89.9 (88.8–90.9)	60	2.0 (1.5–2.5)	118	3.9 (3.2–4.6)	6	0.2 (0.1–0.5)	123	4.0 (3.4–4.8)
**Age**											
**0–4**	639	523	81.9 (78.6–84.7)	32	5.0 (3.6–7.0)	39	6.1 (4.4–8.3)	2	0.3 (0.1–1.3)	43	6.7 (5.0–9.0)
**5–9**	685	620	90.5 (88.0–92.6)	13	2.0 (1.1–3.3)	22	3.2 (2.1–4.9)	0		30	4.4 (3.0–6.3)
**10–14**	1721	1595	92.7 (91.3–93.8)	15	0.9 (0.5–1.5)	57	3.3 (2.5–4.3)	4	0.2 (0.1–0.6)	50	2.9 (2.2–3.9)
**Gender**											
**Female**	1871	1689	90.3 (88.8–91.6)	38	2.0 (1.5–2.8)	69	3.7 (2.9–4.7)	1	0.1 (0–0.03)	74	4.0 (3.1–5.0)
**Male**	1174	1049	89.4(87.4–91.0)	22	1.9 (1.2–2.9)	49	4.2 (3.1–5.5)	5	0.4 (0.2–1.1)	49	4.2 (3.1–5.5)
**Sector**											
**Private**	281	260	92.5 (88.6–95.2)			11	3.9 (2.2–7.1)	1	0.4 (0.1–2.3)	9	3.2 (1.6–6.2)
**Public**	2764	2478	89.7 (88.4–90.8)	60	2.2 (1.7–2.8)	107	3.9 (3.2–4.7)	5	0.2 (0.1–0.5)	114	4.1 (3.4–5.0)

NA- not available

**Table 5:** Treatment initiation status of bacteriologically confirmed rifampicin resistance TB cases.

Variables	Total	Initiated treatment	% (95% CI)	Died	% (95% CI)	Lost to follow-up	% (95% CI)	NA	% (95% CI)
**Overall**	**295**	**257**	**87.1 (82.6–90.6)**	**18**	**6.1(3.8–9.6)**	**14**	**4.8 (2.7–8.0)**	**6**	**2.0 (0.8–4.6)**
**Age**									
**0–4**	42	31	73.8 (57.7–85.6)	6	14.3 (6.0–29.2)	5	11.9 (4.5–26.4)	0	0
**5–9**	66	57	86.4 (75.2–93.2)	4	6.1 (2.0–15.6)	4	6.1 (2.0–15.6)	1	1.5 (0.1–9.3)
**10–14**	187	169	90.4(85.0–94.0)	8	4.3 (2–8.6)	5	2.7 (1.0–6.5)	5	2.7 (1.0–6.5)
**Sex**									
**Female**	176	157	89.2 (83.4–93.2)	11	6.3 (3.3–11.2)	3	1.7 (0.4–5.3)	5	2.8 (1.1–6.9)
**Male**	119	100	84.0 (75.9–89.9)	7	5.9 (2.6–12.2)	11	9.2 (4.9–16.3)	1	0.8 (0.04–5.3)
**Sector**									
**Private**	25	19	76.0 (54.5–89.8)	3	12 (3.6–32.3)	3	12 (3.2–32.3)	0	0
**Public**	270	238	88.2 (83.5–91.6)	15	5.6 (3.3–9.2)	11	4.1 (2.2–7.4)	6	2.2 (0.9–5.0)

NA- not available

### Treatment outcome

Treatment status for 1,164 pediatric TB cases initiated on first-line anti-TB treatment during April 2014 to 15 December 2015 was available at the time of data analysis. Of these, 1,006 (86.4%; CI 84.3–88.3) cases had successfully completed the treatment, 30 (2.6%; CI 1.8–3.7) were still on treatment and 74 (6.4%; CI 5.1–8.0) had died during treatment. A total of 45 (3.9%; CI 2.9–5.2) cases were transferred out to during treatment to their respective RNTCP program management unit, as they were not residents of the same cities. Overall treatment loss to follow-up and failure rates were low: 3 (0.3%; CI 0.1–0.8) and 6 (0.5%; CI 0.2–1.2), patients respectively (Table 6).

**Table 6:** Treatment outcome in rifampicin sensitive cases.

Variables	Grand Total	Completed	% (95% CI)	On Treatment	% (95% CI)	Lost to follow-up	% (95% CI)	Failure	% (95% CI)	Died	% (95% CI)	Transferred out	% (95% CI)
**Grand Total**	1164	1006	86.4 (84.3–88.3)	30	2.6 (1.8–3.7)	3	0.3(0.1–0.8)	6	0.5 (0.2–1.2)	74	6.4 (5.1–8.0)	45	3.9 (2.9–5.2)
**Age**													
**0–4**	214	168	78.5 (72.3–83.7)	9	4.2 (2.1–8.1)	0		0		27	12.6 (8.6–18)	10	4.7 (2.4–8.7)
**5–9**	259	220	84.9 (79.9–89.0)	8	3.1 (1.4–6.2)	0		3	1.2 (0.3–3.6)	17	6.6 (4.0–10.5)	11	4.3 (2.3–7.7)
**10–14**	691	618	89.4 (85–91.6)	13	1.9 (1.1–3.3)	3	0.4 (0.1–1.4)	3	0.4 (0.1–1.4)	30	4.3 (3.1–6.2)	24	3.5 (2.3–5.2)
**Sex**													
**F**	736	644	87.5 (84.8–89.9)	12	1.6 (0.9–3.1)	2	0.3 (0.1–1.1)	6	0.8 (0.3–2)	42	5.7(4.2–7.7)	30	4.1 (2.8–5.8)
**M**	428	362	84.6 (80.1–87.8)	18	4.2 (2.6–6.7)	1	0.2 (0.0–1.5)	0		32	7.5 (5.3–10.6)	15	3.5 (2.0–5.9)

Similarly, 21 TB cases started on second line TB treatment were included for treatment outcomes analysis (treatment initiation during April 2014 to 30^th^ June 2014). Of these 10 (47.6%; CI 26.4–69.7) cases had completed the treatment, 7 (33.3%; CI 15.5–56.9) had died, 2 (9.5%; CI 1.7–31.8) cases transferred out, 1 (4.8%; CI 0.8–22.7) failed treatment and 1 (4.8%; CI 0.8–22.7) was still on treatment. Since the majority of cases were not eligible for treatment outcome, this aspect was excluded from the scope of current manuscript.

### Analysis of case mortality

Of the total 3,340 pediatric TB cases diagnosed under the project, a total of 190 (5.7% CI 4.9–6.5) deaths were observed (148 in rifampicin-sensitive TB cases and 42 rifampicin-resistant TB cases). Of these, 78 (41.1%, CI 34.1–48.4) deaths occurred before treatment initiation and 112 (59.0%, CI 51.6–66.0) deaths were after treatment initiation. Of 112 children who died after treatment initiation, exact date of death could be ascertained for 80 children (Table 7). For these 80 children, 20% (16/80; CI: 12.2%-30.7%) had died within a week of treatment initiation, cumulatively 41.3% (33/80; CI: 30.5–52.8) had died within 15 days of treatment initiation. Overall, 111 of 190 deaths (58.4% CI: 51.1–65.4) had occurred between specimen collection and 15 days of treatment.

**Table 7:** Mortality analysis stratified by treatment initiation of positive cases, age group, sex and past history of treatment.

Variables	Pretreatment mortality, n (%)			On-treatment mortality, n (%)		
	Rif Sen	Rif Res	Total	Rif Sen	Rif Res	Total
**Total**	60 (2.0%)	18 (6.1%)	78 (2.3%)	88 (3.2%)	24 (9.3%)	112 (3.7%)
**Age Group**						
**0–4**	32 (5.0%)	6 (14.3%)	38 (5.6%)	33 (6.3%)	8 (25.8%)	41 (7.4%)
**5–9**	13 (1.9%)	4 (2.1%)	17 (1.9%)	23 (3.7%)	5 (3.0%)	28 (3.5%)
**10–14**	15 (0.9%)	8 (12.1%)	23 (1.3%)	32 (2.0%)	11 (19.3%)	43 (2.6%)
**Sex**						
**F**	38 (2.0%)	11 (6.3%)	49 (2.4%)	51 (3.0%)	13 (8.3%)	64 (3.5%)
**M**	22 (1.9%)	7 (5.9%)	29 (2.2%)	37 (3.5%)	11 (11.0%)	48 (4.2%)
**Past history**						
**No**	58 (2.2%)	18 (9.0%)	76 (2.7%)	71 (3.1%)	18 (11.1%)	89 (3.6%)
**Yes**	2 (0.5%)	0	2 (0.4%)	17 (4.0%)	6 (6.3%)	23 (4.4%)

## Discussion

In spite of recent progress in the development of TB diagnostic tools and available evidence and guidance on their application specifically in the context of pediatric TB and EP-TB, under and delayed diagnosis of childhood tuberculosis remains a major road-block in its effective management [2, 5]. Globally, there remains a major gap between the estimated burden and notification of paediatric TB cases [10, 25]. The current project was undertaken by FIND in coordination with the RNTCP to address this important implementation gap and focused on four major cities of India. This project, dedicated to the pediatric population, offered free of cost, upfront Xpert-based TB diagnosis to all symptomatic presumptive pediatric TB cases from a large number of linked facilities across these cities through a hub-and-spoke model. The current project represents one of largest global efforts exclusively dedicated to the implementation of WHO guidance on the use of upfront Xpert testing for pediatric population and the large project cohort provides useful insights into the potential impact of its effective implementation. The project design ensured rapid specimen transportation, testing, reporting and linkages to treatment and successfully extended routine Xpert testing to large numbers of pediatric specimens, especially non-sputum specimens.

Under the project, application of upfront Xpert testing led to three-fold higher TB detection as compared to smear microscopy along with detection of significant number of pediatric drug-resistant TB cases with a rapid turnaround time. Diagnostic challenges in the management of pediatric TB are well documented [26–29]. Clinical diagnosis in the absence of microbiological confirmation has been relied on extensively over the years for the diagnosis of TB in children, however clinical symptoms in children are often non-specific due to which TB diagnosis is complicated and/or delayed [2, 10–11]. Different clinical definitions used for diagnosis of TB in children have shown inconsistent and variable performance in different settings [13]. These factors often led to significant TB diagnostic delay in children, contributing to TB related morbidity and mortality [5,26]. Further diagnosis and management of rifampicin-resistant TB in the absence of laboratory confirmation poses a major challenge in this highly vulnerable age-group [5]. The findings from the current project demonstrate the ability of a rapid diagnostic assay in effectively addressing these diagnostic challenges in presumptive pediatric TB cases.

TB detection rates in female pediatric presumptive TB cases was two-fold higher as compared to males. Similar findings have earlier been earlier from India and Afghanistan [30,31]. This finding could not be attributed to variations in age, prior history of TB treatment or sectoral variations. While no clear explanation of this finding could be projected from the project data, it seems probable that a combination of different gender-related factors like access to health care may be part of the explanation. Thus it is not known whether this is a genuine difference in incidence or the result of gender variation in patient pathways or a combination of both.

More than half of the samples tested under the project were non-sputum specimens. Xpert performance on both sputum and non-sputum specimens provided a high proportion of valid results, similar to earlier reports [32–35]. The project implemented over two years in uncontrolled settings, demonstrates the feasibility of routinely applying Xpert testing to both sputum and non-sputum specimen. TB and DR-TB detection rates were similar in both sputum and non-sputum specimens. High TB positivity was observed in most specimens especially gastric aspirates/lavage, BAL, pus, lymph node, and pericardial fluid. Further, the project data clearly demonstrates limited utility of subjecting non-sputum specimen to smear microscopy. Xpert positivity rates on pleural fluid and ascitic fluid in the current study were very low, with limited additional gain as compared to smear microcopy, in line with WHO guidance and similar other studies [16,36]. A large number of rifampicin-resistant TB cases would have been missed in the absence of Xpert testing of non-sputum specimens, especially cases of rifampicin-resistant TB meningitis. These findings also have relevance in the context of adult extra-pulmonary TB (EP-TB) cases where higher levels of drug resistance are likely to be observed as compared to a pediatric population.

Under the project we observed alarmingly high levels of rifampicin-resistance in the diagnosed TB cases in the enrolled cohort of presumptive pediatric TB cases. The levels were high across the four cities, and didn’t show any clustering around particular facilities or time period or sector (public or private). Levels of rifampicin resistance were high in all three pediatric sub-age-groups. The majority of the DR-TB cases were smear negative and had no past history of previous TB treatment. Resistance levels were higher in cases with history of past TB treatment; however, the majority of the rifampicin-resistant cases were treatment naïve. While the project cohort wasn’t drawn purposively to capture a representative sample/estimate of children in India or these cities, levels of rifampicin-resistant TB observed in this large cohort is of great concern and suggests high levels of ongoing transmission. Children are not a priority target group for DR-TB control interventions, with the majority of pediatric TB cases being clinically diagnosed in the absence of microbiological confirmation. There is no systematic routine monitoring and reporting of rifampicin resistance in the pediatric population. The levels of rifampicin resistance observed in this project suggest a need for further research in this area and routine monitoring of DR-TB in pediatric population, both from programmatic management and epidemiological perspective [24,37]. Further, specimen-wise analysis showed that more than half of the rifampicin-resistant results were observed in non-sputum specimens. Amongst the sputum specimens, more than half of the rifampicin-resistant results were observed amongst specimen which were negative on smear microscopy. Such specimens are generally not subjected to upfront drug susceptibility testing in routine practice and such cases are either managed clinically or diagnosis is based on histopathological examination, which potentially can lead to missed diagnosis of drug-resistant TB, leading to sub-optimal TB treatment.

Under the project, in cases of high clinical suspicion and negative test result on Xpert, treating providers were encouraged to have additional patient specimens tested. Significant gains in the diagnostic yield on testing a second patient specimen were observed; however, on testing the third specimen the gain in diagnostic yield was only marginal. Similar other studies have also reported gains in sensitivity on testing an additional patient specimen [34, 38].

Since the outset of the project, same day diagnosis was one of the key project components. This was pertinent as the project was being implemented in the largest cities of the country, requiring turnaround time to be regularly monitored in the patients’ interest. Rapid turnaround time to test result was achieved by synergy of interventions such as local consultative approach in developing same day transportation linkages between the city labs and linked facilities utilizing services of volunteers, having high throughput labs to accommodate any intra-day workload surges, provision of having need based extended working hours, and 100% electronic reporting of results. This rapid diagnostic time in turnaround facilitated prompt treatment initiation both for rifampicin-sensitive and resistant TB cases. Availability of lab results with upfront information on rifampicin susceptibility enabled prompt initiation of appropriate treatment regimens, effectively addressing the diagnostic challenges faced by treating providers, minimizing the need for subjectively interpreting clinical presentations of the disease, thereby simplifying the patient pathway bottlenecks and delays which have been flagged in various studies [39–42]. Infield implementation of the project design over two years in the largest cities of India demonstrates the feasibility of replicating the project design in other similar and even smaller settings.

Quick turnaround time to test results and upfront availability of information on rifampicin susceptibility, leading to appropriate and rapid treatment initiation contributed to high treatment success rates. Overall loss to follow-up and treatment failure rates were exceptionally low (less than 1%).

However, in spite of rapid diagnostic time and prompt treatment initiation, significant levels of mortality were also observed in the project cohort. Mortality rates were higher in the 0–4 years, followed by 5–9 years and 10–14 years age-group. Review of overall mortality in the project cohort of diagnosed cases revealed that more than 5% of the children diagnosed with TB died (before or during treatment). In spite of the very quick diagnostic turnaround time and prompt access to treatment, it was concerning to see majority (58%) of these deaths occurring within two weeks of diagnosis. High proportion of mortality occurring early in the course of patient care suggests either patient health seeking delays or provider associated delays in prescribing free of cost Xpert testing which needs to be better understood and addressed. Similar, high mortality rates were observed in previous studies and suggested the need to intensify efforts at improving notification and treatment outcomes in children [43,44]. Other studies have also suggested that major advances needs to be made in the understanding of the epidemiology, diagnosis and treatment of childhood TB [45]. In this regard, a separate study on the pediatric patient pathways was undertaken as part of this project which will likely provide more insight on this aspect.

## Conclusion

The current project implemented over more than two years demonstrated the feasibility of rolling out rapid and upfront Xpert testing for pediatric presumptive TB cases. The project design of a single Xpert lab per city efficiently covered each of the four large cities of India. The hub and spoke model implemented as part of the project ensured a rapid turnaround testing time, which in turn facilitated prompt and appropriate treatment initiation. These results further suggest that the upfront Xpert assay is a promising solution to address TB diagnostic challenges in children, testing both sputum and non-sputum specimens. The high levels of rifampicin resistance detected in pediatric TB cases, while being a major cause of concern from epidemiological prospective, also underscores the need to further prioritize upfront Xpert access to this vulnerable population.

## Supporting information

S1 Data Supporting data file- compiled sheet till 30 Jun16_23-1-18.xlsb. (XLSB)
